# Thoracoscopic Congenital Esophageal Stenosis Repair and Thal Fundoplication by Right Thoracic Approach: A Case Report

**DOI:** 10.70352/scrj.cr.24-0180

**Published:** 2025-04-07

**Authors:** Kazuya Nagayabu, Wataru Sumida, Kazuki Ota, Yasuyuki Ono

**Affiliations:** Department of Pediatric Surgery, Aichi Children’s Health and Medical Center, Morioka-cho, Obushi, Aichi, Japan

**Keywords:** congenital esophageal stenosis, thoracoscopic radical esophagectomy, thoracoscopic fundoplication, right thoracic approach

## Abstract

**INTRODUCTION:**

Congenital esophageal stenosis (CES) is a rare clinical condition found in 1 in 25000–50000 live births. Surgical treatment is required when endoscopic balloon dilatation is ineffective. Laparoscopic and thoracoscopic approaches are selected based on lesion location. Gastroesophageal reflux (GER) is often observed as a postoperative complication that necessitates additional fundoplication. We report a case of CES in the lower third of the esophagus that was treated with simultaneous thoracoscopic resection and Thal fundoplication using the right thoracic approach.

**CASE PRESENTATION:**

The patient was a 6-month-old boy who presented with vomiting after consuming solid food. Although he had been previously treated by a physician, he was referred to our hospital for further examination because of persistent symptoms at 1 year and 7 months of age. As his oral intake was insufficient, he was thin compared with his twin brother. On esophagography, an abruptly narrowing lesion was found at the Th9-10 level, and congenital esophageal stenosis was diagnosed. Since balloon dilatation under upper gastrointestinal endoscopy was ineffective, the patient was treated surgically. Thoracoscopic esophagectomy (end-to-end anastomosis) and fundoplication (Thal procedure) were simultaneously performed via the right thoracic cavity. Although transient postoperative gastric paresis due to vagus nerve injury was observed, the patient improved with medical treatment and was discharged on postoperative day 14. He is currently able to ingest solid food orally, without GER.

**CONCLUSIONS:**

CES can be a surgical indication for a thoracoscopic approach, depending on the site of the lesion. This is the first case in which anti-reflux surgery was performed simultaneously with thoracoscopic CES repair. We consider that this technique is useful for preventing not only GER, but also anastomotic leakage.

## Abbreviations

CES
: congenital esophageal stenosis
EBD
: endoscopic ballon dilatation
GER
: gastroesophageal reflux
POD
: postoperative day
SD
: standard deviation
TBR
: tracheobronchial remnants

## INTRODUCTION

Congenital esophageal stenosis (CES) is a rare clinical condition found in 1 in 25000–50000 live births. The symptoms of CES usually include dysphagia after the introduction of semisolid alimentation. Endoscopic balloon dilatation (EBD) is the first-line treatment of CES.^1–3)^ If balloon dilatation does not relieve the patient from symptoms, surgical treatment should be considered.^1–3)^ In recent years, endoscopic surgery has become more common as a surgical treatment, and in such situations, a decision must be made as to whether the laparoscopic or thoracoscopic approach is appropriate depending on the location of the lesion.^4,5)^

If a lesion is present in the lower esophagus, postoperative gastroesophageal reflux (GER) is likely to occur. Its mechanism seems to be related to a decrease in lower esophageal sphincter tone and shortened length of the intraabdominal esophagus, secondary to the overall shortening of the esophagus after esophagectomy.^1)^

We encountered a case of a CES located immediately above the diaphragm. We considered that the thoracoscopic approach was preferable for treating the lesion because of its location. Furthermore, simultaneous anti-reflux surgery was required to prevent postoperative complications.

There are a few reported cases of CES repair with simultaneous fundoplication even when the lesion was located in the abdominal esophagus. Here, we report the first case of CES with simultaneous thoracoscopic resection and fundoplication.

## CASE PRESENTATION

A 19-month-old boy was referred to our hospital due to failure to thrive. The patient was born as the first twin via emergency caesarean section because of premature rupture of the membranes. Although his birth weight was 1702 g (−2.5 standard deviations [SD]), he was gaining weight steadily with only milk intake. He started vomiting after feeding at approximately 6 months of age and had difficulty ingesting age-equivalent meal forms. At the age of 10 months, he consulted a primary care physician who allowed him to consume only a shredded diet and fluids, including milk. However, his symptoms did not improve. Consequently, his growth became impaired over time. The patient was referred to our hospital for further evaluation and treatment.

**Fig. 1:**
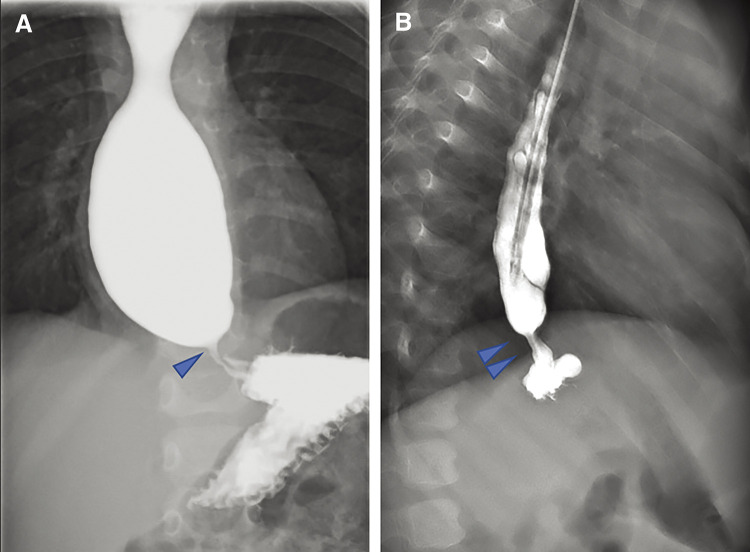
Esophagogram findings. (**A**) Anterior view of the esophagogram. Arrowhead indicates the stenotic lesion of the lower esophagus. Oral side of the stenotic lesion was dilated. (**B**) Lateral view of esophagogram. The stenotic lesion was found at the Th9-10 level and above the diaphragm (arrow).

On the initial visit to our hospital, his height was 76 cm (–1.9 SD) and his weight was 8.08 kg (–1.9 SD). Based on the patient’s clinical course, we initially suspected GER or CES. Upper gastrointestinal imaging revealed a stenotic lesion in the esophagus at the level of Th 9 or 10, but no GER was observed (**Figs. 1A**,**1B**). The upper gastrointestinal imaging results indicated that his symptoms were due to CES. The patient was treated with only a single balloon dilation during upper gastrointestinal endoscopy. However, this intervention proved ineffective, with no improvement in the patient's symptoms. Therefore, surgical intervention was required.

As the stenotic lesion was considered to be on the cranial side of the diaphragm, we determined that a thoracoscopic approach was preferable. Furthermore, because of the high probability of postoperative GER, we believed that anti-reflux surgery should be performed simultaneously.

**Fig. 2:**
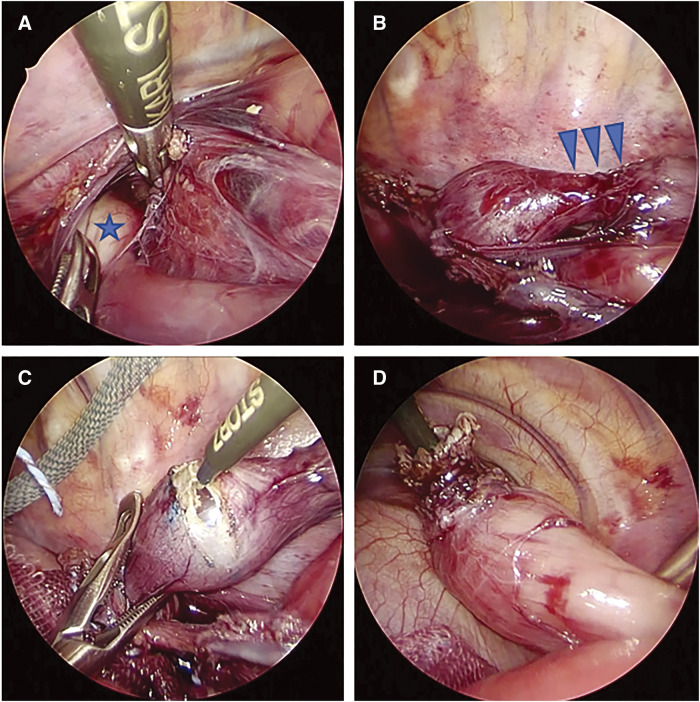
Pulling of the gastric fundus to the thoracic cavity and resection of the stenotic lesion of the esophagus. (**A**) The gastric fundus (blue star) was found via the diaphragm after resection of the diaphragmatic-esophageal ligament. (**B**) With a balloon catheter, the range of the lesion (blue arrow) was identified easily. (**C**, **D**) Incision along the balloon can minimise the length of resection of the esophagus.

**Fig. 3:**
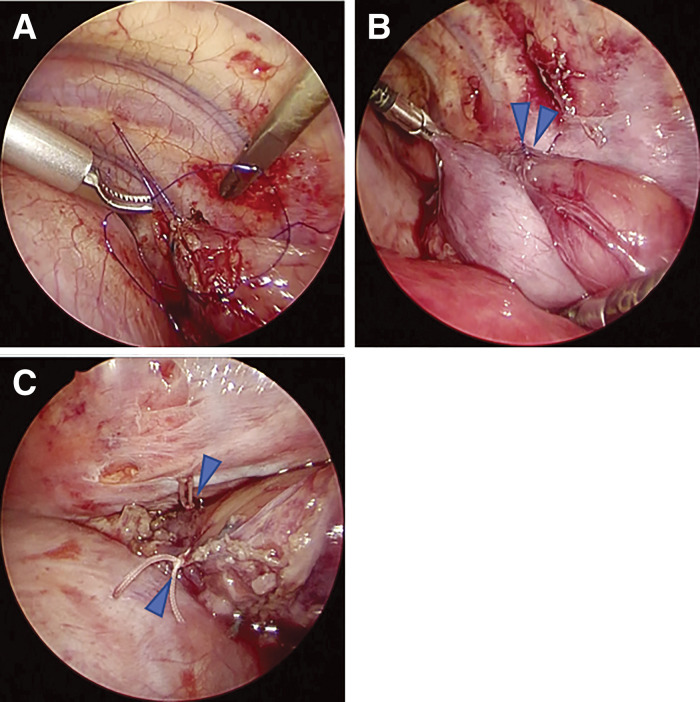
End-to-end anastomosis and Thal fundoplication. (**A**) Anastomosis of the esophagus. All layers were sutured in a single layer with 5-0 PDS. (**B**) The gastric fundus was wrapped over the anterior wall of the esophagus, including the anastomosis (blue arrow). (**C**) The esophagus was fixed with the diaphragmatic crus (blue arrow).

Thoracoscopic surgery was performed using four ports in the left half-supine position with one-lung intubation using a blocker under general anaesthesia. After dissecting the diaphragmatic-esophageal ligament to reach the abdominal cavity, we located the gastric fundus via the diaphragm (**Fig. 2A**). The dilated esophagus was easily identified, and the range of the stenotic lesion was confirmed with a balloon catheter to minimise the length of the esophageal resection (**Figs. 2B**–**2D**). The vagus nerve was removed from the mediastinal side. The stenotic lesion of the esophagus was resected and anastomosed in an end-to-end fashion with 5-0 polydioxanone (PDS) (**Fig. 3A**). Subsequently, the gastric fundus was wrapped over and fixed to the anterior wall of the esophagus with three sutures of 2-0 ETHIBOND (Johnson & Johnson Med Tech, New Brunswick, NJ, USA), and the esophageal anastomotic site was covered with the gastric wall (Thal fundoplication) (**Fig. 3B**). The anastomosis with half-gastric wrapping was returned to the abdominal cavity after esophageal traction was released. The esophageal wall and diaphragmatic crus were fixed using 2-0 ETHIBOND (Johnson & Johnson Med Tech) (**Fig. 3C**).

**Fig. 4:**
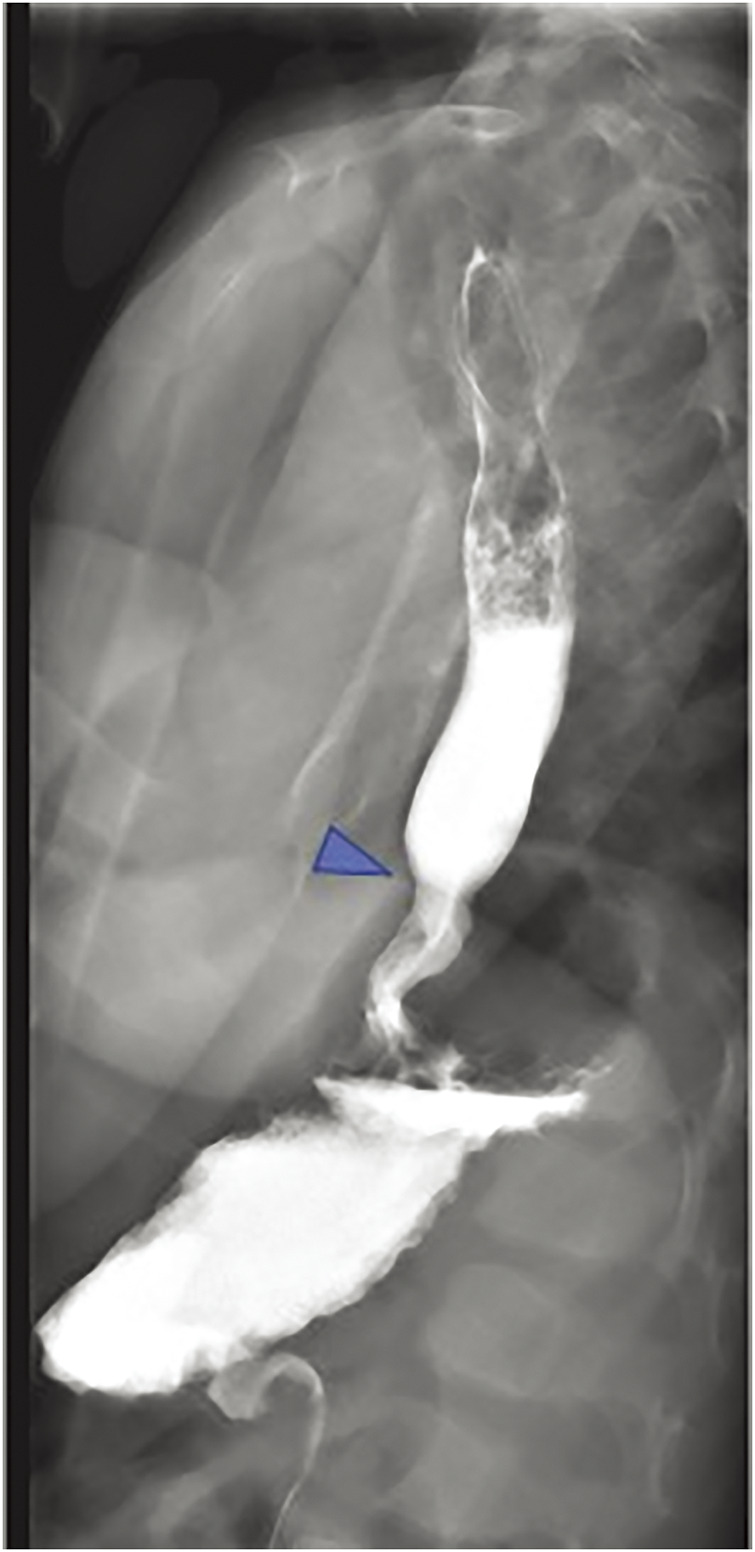
Postoperative esophagogram findings. We performed esophagogram on the patient 1 year after surgery to estimate the passage. Although mild anasmotic stenosis was seen, contrast medium can pass through without delay.

Postoperatively, the patient remained intubated with muscle relaxants for 1 day in the paediatric intensive care unit, and enteral feeding was started on postoperative day (POD) 3. Esophagography performed on POD 7 confirmed no signs of anastomotic failure or GER. Postoperative radiography revealed distension of the stomach, which was considered to be gastric paralysis due to vagal nerve injury. Therefore, a nasogastric tube was inserted for gastric decompression, and rikkunshito and mosapride were administered. The stomach distension promptly improved after treatment, and the patient was discharged with full oral feeding on POD 14. Currently, no significant passage obstruction was observed, despite mild stenosis at the anastomotic site with upper gastrointestinal imaging 1 year postoperatively (**Fig. 4**).

**Fig. 5:**
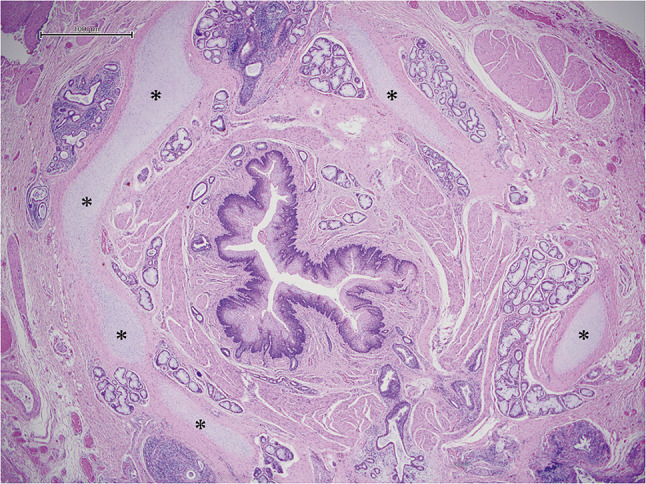
Pathological findings. Cartilage components are illustrated with an asterisk. Pathological examination found that cartilage components surround three-quarters of the esophagus, which are consistent with CES by tracheobronchial remnants.

Pathological examination revealed cartilage components surrounding three-quarters of his esophagus, and a diagnosis of CES due to stenosis with a tracheobronchial remnant (TBR) was made (**Fig. 5**).

## DISCUSSION

CES can be classified into three histological types: tracheobronchial remnants, segmental fibromuscular hypertrophy, and membranous stenosis.^1–3)^ The treatment strategy for CES is to first attempt EBD, followed by surgical resection if endoscopic dilation is unsuccessful.^2,3)^

Laparoscopic or thoracoscopic resection is selected as the surgical approach depending on the site of the lesion.^4–9)^ In the present case, the lesion was located in the lower thoracic esophagus, just above the diaphragm. Therefore, a thoracoscopic approach via the right thoracic cavity was preferred in this case.

As a postoperative complication, GER is expected to result from a dull His angle provoked by shortening of the esophageal length.^3,10)^ We consider that controlling for this complication is an important factor for the success of surgery for CES. Laparoscopic fundoplication is performed by several surgeons. There are some case reports on the sequential performance of laparoscopic CES resection and fundoplication.^4,10)^ Moreover, some reports have proposed that simultaneous laparoscopic fundoplication for the resection of CES in the distal esophagus should be performed, not only to prevent anastomotic leakage, but also GER.^1,4)^ Despite the absence of explicit mention in the cited references regarding the impact of the anti-reflux procedure on the anastomosis, it is hypothesized that the fact that the anastomotic site is surrounded by the gastric wall, contributes to the effectiveness of conservative treatment, even in cases of postoperative anastomotic failure. Although there are many ways of fundoplication, the reason why we chose the Thal procedure is that it is a simple procedure that is easy to manipulate and we expected that it would not interfere with his postoperative oral intake.

However, no reports on simultaneous thoracoscopic fundoplication and CES resection have been published. Although the site of the lesion in our case was located in the thoracic cavity, the anastomosis was expected to be near the esophagogastric junction, according to the preoperative imaging. Therefore, we assumed that our patient had a risk of postoperative GER comparable to that in an intraabdominal case. We thus consider that our simultaneous procedure is reasonable and desirable. Also, regarding the indication for this procedure, our patient was of typical age for CES, but was a twin and smaller than usual. Despite his small stature, we were able to perform this operation without major problems, and we consider that this technique can be adapted to a wide range of CES patient populations.

Other surgical procedures for CES have been reported.^4,5,7)^ For example, there are some reports of esophagoplasty, such as partial resection or myectomy, even in cases of CES with TBR as in our case^5,7)^; however, we selected complete resection for this case. This is because other studies have reported that left epithelial and glandular tissue after partial resection can be the cause of stenosis, and complete resection is recommended, especially in CES cases of the TBR type.^10)^ The present case did not undergo endoscopic ultrasonography. However, the morphology of the stenosis on preoperative imaging and the ineffectiveness of balloon dilation allowed for a preoperative prediction of TBR type.

Postoperative gastric paralysis due to vagus nerve injury was observed in this case, but gastric tube decompression and oral administration of rikkunshito and mosapride quickly improved the paralysis. Generally speaking, this nerve paralysis is considered to be one of the most common complications of esophagectomy. We consider that the surgical maneuver caused transient paralysis of the vagus nerve, even though energy devices were not used when the vagus nerve was transected from the esophagus.

## CONCLUSIONS

We performed radical thoracoscopic repair and Thal fundoplication simultaneously in a patient with CES. We consider that this new procedure can be effective in reducing complications by using a thoracoscopic approach for CES.

## ACKNOWLEDGMENTS

The language used in this manuscript has been reviewed by Editage.

## DECLARATION

### Funding

None.

### Authors' contributions

KN wrote the manuscript.

WS revised and approved the manuscript.

KN and YO performed surgeries.

All authors were involved in the postoperative management.

All the authors commented on the manuscript and approved its final version.

### Availability of data and materials

Not applicable.

### Ethics approval and consent to participate

This case report was approved by the ethics committee and the director of our hospital.

### Consent for publication

The patient provided informed consent for the publication of this report.

### Competing interests

The authors declare no competing interests with respect to this report.
